# Challenging Situations in High and Intensive Psychiatric Care: Professional Perspectives and Key Characteristics

**DOI:** 10.3390/nursrep16100356

**Published:** 2026-10-09

**Authors:** Isa C. de Jong, Sylvia Gerritsen, A. Laura van Melle, Cornelis L. Mulder, Mariëtte A. van den Hoven, Yolande Voskes

**Affiliations:** 1 Department of Ethics, Law and Humanities, Amsterdam University Medical Centers, 1081 HV Amsterdam, The Netherlands; s.gerritsen1@amsterdamumc.nl (S.G.); l.vanmelle@ggzingeest.nl (A.L.v.M.); m.a.vandenhoven@amsterdamumc.nl (M.A.v.d.H.); y.voskes@amsterdamumc.nl (Y.V.); 2 GGZ Ingeest, 1081 JC Amsterdam, The Netherlands; 3 Department of Psychiatry, Epidemiological and Social Psychiatric Research Institute, Erasmus MC, 3015 GD Rotterdam, The Netherlands; niels.clmulder@wxs.nl; 4 Parnassia Psychiatric Institute, 3066 TA Rotterdam, The Netherlands; 5 Mental Healthcare Centre GGZ Eindhoven, 5626 ND Eindhoven, The Netherlands; 6 Tilburg School of Social and Behavioral Sciences, Tranzo Scientific Center for Care and Welfare, Tilburg University, 5037 DB Tilburg, The Netherlands

**Keywords:** psychiatric nursing, qualitative research, challenging situations, acute psychiatric care, health care teams

## Abstract

**Background**: Managing acute psychiatric crises remains a major challenge, as some situations exceed the capacity of even specialised care. These situations typically arise from a complex interplay of patient, team, ward and organisational factors, leading to prolonged admissions, increased use of coercive measures, and distress for both patients and professionals. **Aims**: This study aimed to identify the most challenging situations within High and Intensive Care wards, clarify their multifactorial origins, and explore strategies for more effective management. **Method**: This multi-centre, multi-method sequential qualitative study involved two phases: collecting 40 challenging cases, synthesized into two fictional vignettes, and discussing these in three focus groups with 33 mental health professionals, with data analysed through qualitative content and thematic analysis. **Results**: Participants recognised the vignettes and emphasised that it is the combination of patient, team, ward and system factors that places teams under sustained pressure, undermining the therapeutic environment and safety. These situations often evoke strong emotional responses and certain team dynamics, such as countertransference and splitting. Key challenges included dilemmas around admission, lack of treatment perspective, and systemic barriers. Suggested strategies focused on reward-based and autonomy-promoting care, requiring improved team communication, decisive clinical decision-making, and shared responsibility with external partners. **Conclusions**: Addressing challenging situations in High and Intensive Care wards calls for timely recognition, open discussion, structured team reflection, and ongoing staff training. Personalised care, clear protocols, and robust cross-sector collaboration are essential. Ongoing evaluation and further research, including patient and network partner perspectives, are needed to optimize interventions and ensure effective, empathic care.

## 1. Introduction

Mental health institutions aim to provide optimal care for individuals in psychiatric crisis when home-based support is insufficient, developing clinical models such as High and Intensive Care (HIC) in the Netherlands to serve severely ill patients at high risk of harm [1]. However, professionals increasingly encounter complex and disruptive situations that stretch the limits of safe and effective management [2,3,4]. These situations often involve extreme aggression, self-harm, or persistent disruptive behaviour, resulting in ineffective treatment, prolonged admissions, and high rates of restraint and medication use, as well as emotional and physical distress for care teams [2,5,6,7]. Exposure to severe aggression can lead to anxiety, depression, post-traumatic stress disorder (PTSD), and even fatal outcomes [7]. In acute psychiatric care, and in the HIC model in particular, establishing and maintaining contact with the patient is essential. As nurses provide continuous, round-the-clock care and form the largest part of the team, they are most directly involved in this contact. While this makes them frequent targets of aggression, it is precisely this proximity that also makes them key agents of change, uniquely positioned to recognise early warning signs, de-escalate tension, and prevent challenging situations.

Understanding and preventing such situations is therefore a core concern for mental health nursing. One of the earliest studies on this topic, published in 1979, used the term “difficult patients” and already recognised that these challenges are multifactorial [8]. More recent studies increasingly confirm that challenges arise from a dynamic interplay between patient, team, and organisational factors [2,3]. Despite this, there is still a significant gap in research on which specific factors drive challenging situations, how they interact in daily practice, and how care teams can most effectively address them in acute inpatient psychiatric care.

## 2. Background

### 2.1. HIC Model

Introduced in 2013, the HIC model set a new standard for acute psychiatric wards in the Netherlands, aiming to prevent crisis escalation, reduce coercion, and promote recovery-oriented care [1]. Central to HIC is a care ethics approach, emphasising empathy, attentiveness, and relational engagement. The model focuses on restoring contact and patient autonomy, rather than relying on a purely biomedical approach and solitary confinement. HIC Patients are in severe psychiatric crisis, at significant risk of losing autonomy and/or causing harm, and for whom outpatient care is insufficient [9]. The model uses a stepped-care principle, starting with crisis prevention and early intervention by ambulatory teams ((F)ACT, IHT, Crisis Intervention), who remain involved during admission [1]. Admissions are brief and aim to help patients regain control, enabling them to resume daily life outside the HIC. The ward itself is designed as a healing environment, in which physical design and atmosphere support recovery. Care in the High Care (HC) area centers on group-based stabilization and recovery. If a patient becomes increasingly distressed or group care is no longer safe, intensive support can be provided in their own room. This approach combines close guidance and clear boundaries to help the patient feel safer and more settled, supporting their return to the group environment. If, despite these efforts, safety for the patient, staff, or visitors due to severe risk of harm, cannot be guaranteed, care is escalated to the Intensive Care (IC). The IC offers one or more Intensive Care Units (ICUs), each comprising at least a bedroom and private bathroom. These rooms allow for one-to-one care and personalised attention, enabling patients to regain their calm and begin to restore self-regulation. If even intensive support in the IC is insufficient to ensure the safety of the patient or others, patients are, as a last resort, transferred to the High Security Room (HSR) for close monitoring. Importantly, the HSR is not a treatment intervention but solely a safety intervention: a specially designed, highly secure room with private bathroom and smart home technology to maximize autonomy and comfort. Its use is considered a coercive intervention, is strictly supervised, and is kept as brief as possible.

### 2.2. Defining Challenging Situations

There is no single definition of “challenging situations” in acute psychiatric care. Most studies refer to the definition of “challenging behaviour” by the Royal College of Psychiatrists [10,11], which describes it as actions of such intensity, frequency, or duration that threaten quality of life or safety and often lead to restrictive or exclusionary responses. This includes aggression, problematic sexual behaviour, self-injury, destructiveness, and other stereotypical behaviours shaped by organisational, regulatory, and societal factors [4,5,12]. Other studies emphasise the perceived danger of these behaviours [13], and highlight the fear and distress they can evoke in such “dangerous situations” [14]. Other terms in the literature include “challenging situation” for cases that hinder high-quality care [15], “care disruption” for severe ward disturbances [2], and “refractory cases” for situations where standard interventions fail [3]. In this study, we use “challenging situations” to emphasise the complex interplay of patient, team, and organisational factors, and the need for a constructive, solution-oriented approach.

### 2.3. Aim

More than a decade after the implementation of the HIC model, professionals continue to encounter complex situations in which regular care processes may become disrupted or prove insufficient [2,3,16]. Recent studies have described complex and refractory situations in which regular psychiatric care reaches its limits [3] and have explored approaches to challenging behaviour in acute psychiatric wards [12], underscoring the urgent need for research into effective solutions in inpatient acute psychiatry. Therefore, this study aims to systematically identify the types of situations that most challenge inpatient care teams at HIC wards, explore their underlying dynamics, and clarify what is required to effectively address them. Accordingly, this study addresses the following two-part research question: What characterizes challenging situations at HIC wards, and what do care professionals consider necessary for effectively dealing with these situations?

## 3. Methods

### 3.1. Design

This study used a multi-centre, multi-method sequential qualitative design as part of a national research project on HIC in the Netherlands. The current research consisted of two phases: 1. case study collection and development of vignettes, and 2. focus groups to discuss the vignettes. By combining data from different sources (case reports and group discussions) and using multiple qualitative methods, this approach enabled methodological triangulation, thereby strengthening the reliability and validity of the findings [17].

### 3.2. Wards and Participants

All mental health institutions in the Netherlands that operated an HIC unit were invited to participate, with 24 institutions ultimately joining the study. Each institution selected one HIC ward, with the inclusion criterion of providing adult psychiatric care (18+). Each ward appointed contact persons to liaise with the research team. In Phase 1, the contact persons were asked to collect two challenging cases from their ward, either from their own practice or from that of colleagues. In total, cases were received from 20 wards, while 4 wards did not submit any. In Phase 2, a purposive sampling strategy was applied for the focus groups: the contact persons of the 24 participating wards were asked to recruit 2 professionals per ward, preferably from different disciplines, to achieve maximum variation in professional backgrounds [17]. In total, 21 wards were represented in 3 focus groups; 3 wards did not participate due to insufficient capacity. Participants (*n* = 33) included 22 (coordinating) nurses, 4 peer workers, 2 social workers, 2 psychiatrists, 2 team leaders and 1 activity counsellor.

### 3.3. Data Collection

Data were collected between March 2023 and March 2024. In Phase 1, challenging cases were collected using a structured, open-ended case inquiry (Table 1) to ensure consistency. After piloting the format and system with 3 wards, the contact persons submitted a total of 40 cases securely and anonymously via the online Castor Electronic Data Capture software (Ciwit B.V., Amsterdam, The Netherlands) to reduce bias [17]. As each participating ward belonged to a different institution, the case inquiries were completed independently of one another, and the researchers could not trace which ward had submitted which case. The submitted cases therefore represent situations that HIC professionals themselves perceived as most challenging, in line with the aim of this study. The cases were analysed and translated into two fictional vignettes reflecting the two types of challenging situations identified (see Data Analysis; Box 1 and Box 2). In Phase 2, 3 focus groups were held at Amsterdam University Medical Centers, each lasting 90 min. The sessions were moderated by authors 2, 3, and 6, assisted by an independent researcher who had been involved in the case analysis. The vignettes were presented and discussed using a semi-structured interview guide (Table 2). All focus groups were audio recorded and transcribed verbatim. Written informed consent was obtained from all participants.

### 3.4. Analysis

In Phase 1, the 40 submitted cases were analysed using qualitative content analysis, combining deductive and inductive coding [17]. The structured format of the case inquiry (Table 1), distinguishing patient-, team-, and ward-/system-related factors, served as an a priori coding framework. Author 1 coded the cases accordingly. To enhance objectivity, an independent researcher was involved, and both independently reviewed the first 12 cases. Through investigator triangulation [17], they compared and discussed their findings. Despite some variations in details, the cases clustered into two distinct types of challenging situations, comprising 28 and 12 cases, respectively. This distinction emerged from the analysis rather than being predefined: across the independently submitted cases, the same combinations of patient-, team-, ward- and system-related factors recurred, and these factors proved closely interrelated. The possibility of additional types was explored, but no consistent third combination of factors emerged. As the cases were submitted independently and anonymously by different wards, this convergence across independent sources supports the validity of the two identified types. Each type was developed into a fictional vignette (Box 1 and Box 2) by combining the most common factors, while omitting case-specific details to ensure anonymity. A summary of all cases, including their classification, is provided in the Supplementary Table S1.

For Phase 2, the focus group data were analysed thematically using open, axial, and selective coding [17] in MAXQDA (version 24; VERBI Software, Berlin, Germany). Again, investigator triangulation was performed. Authors 1 and 2 independently coded the first focus group, compared codes, and reached consensus. Author 1 continued open coding for the remaining groups, after which both authors clustered codes into themes (axial coding) and identified overarching categories (selective coding). All three focus groups were conducted before analysis commenced; saturation was therefore assessed retrospectively during analysis. When the codes of the three focus groups were systematically compared, the codes identified in the first group recurred in the subsequent groups, and the third group yielded no new codes or themes; at this point, data saturation was considered achieved [17]. As the same two vignettes were discussed in each focus group, the convergence of reflections across groups further supported saturation.

#### 3.4.1. Ethics Statement

The authors assert that all procedures contributing to this work comply with the ethical standards of the relevant national and institutional committees on human experimentation and with the Helsinki Declaration of 1975, as revised in 2024 [18]. The Medical Ethics Review Committee of the Amsterdam University Medical Centers concluded that this study is not subject to the Dutch Medical Research Involving Human Subjects Act (nWMO; reference number 2022.0625). The study was conducted and reported in line with the COREQ (Consolidated Criteria for Reporting Qualitative Research) guidelines and a completed COREQ checklist has been added as Supplementary Material S1.

#### 3.4.2. Reflexivity Statement

The research team consisted of a PhD student with a background in nursing and health sciences; a researcher with a PhD in forensic high and intensive care (FHIC); a researcher with a PhD in high and intensive care (HIC), who also worked as a coercion and restraint coordinator; a professor of psychiatry; a professor of ethics; and an assistant professor specialised in the reduction in coercion. This diversity in backgrounds facilitated critical reflection on the data from multiple perspectives, reducing the influence of individual assumptions on the analysis and interpretation. Authors 3 and 6 were professionally affiliated with two of the participating mental health institutions and, through their professional roles, were acquainted with some of the focus group participants, although they did not work on the participating HIC wards and were not members of the clinical teams involved. The remaining researchers had no such affiliations. Coding and interpretations were subsequently discussed within the research team, with discrepancies resolved through consensus.

## 4. Results

### 4.1. Vignettes

Based on the analysis of the 40 submitted cases, two distinct types of challenging situations were identified. Each type was characterized by a specific, recurring combination of patient, team, ward and system factors, and was translated into a fictional vignette (Box 1 and Box 2). Vignette 1 describes a 36-year-old man with schizophrenia, mild intellectual disability (MID) and antisocial behaviour, admitted under a compulsory measure following threats towards his family. His admission is marked by repeated absconding, substance use, and serious aggression, while collaboration with the police proved challenging and no clear treatment or risk management plan is in place. Vignette 2 describes a 20-year-old woman with post-traumatic stress disorder and personality and dissociative disorder, whose suicidality and self-harm escalate during involuntary admissions. Trauma therapy has repeatedly been discontinued, the team is divided over continued HIC admissions, and the outpatient team is unable to provide care in the home environment. Both vignettes were presented to the focus groups in Phase 2.

Box 1 **Vignette 1.**

A 36-year-old man has been admitted to the High and Intensive Care (HIC) unit under a Compulsory Measure (CM) following threats made towards his family with a knife. The patient has a history of multiple admissions within mental health and addiction services. He is diagnosed with schizophrenia, mild intellectual disability (MID), and displays antisocial behaviour. In recent weeks, the patient has repeatedly absconded during supervised leave, during which he has engaged in excessive substance use, including cocaine, heroin, cannabis, and alcohol. This culminated in a life-threatening situation that required emergency department intervention. During the current admission, several serious incidents of aggression have occurred, including an attempted strangulation of a staff member. Collaboration with the police during these incidents proved challenging. The patient’s behaviour evokes countertransference within the team, provokes aggression from others, and he has sought sexual contact, whether consensual or not, with fellow clients. On the ward, he actively seeks access to drugs and alcohol and attempts to supply these to other clients. There is currently no clear treatment or risk management plan in place. Additionally, frequent changes within the team have led to a lack of cohesion and effective collaboration among staff members. The patient has no fixed address and is not accepted into supported living arrangements or high-security settings.

Box 2 **Vignette 2.**

On Friday evening, a 20-year-old woman is announced for admission to the High and Intensive Care (HIC) unit. She has a psychiatric history of Post-Traumatic Stress Disorder (PTSD), personality and dissociative disorder. She has been regularly admitted, often involuntarily, due to suicidality and self-harming behaviour, which tends to escalate during inpatient stays. The patient has a background of early childhood trauma, for which no completed trauma treatment has yet taken place. Each time trauma therapy is initiated, suicidal behaviour and dissociative episodes intensify, leading to the discontinuation of treatment, most recently as well. There is significant countertransference within the team, primarily due to the severity of the self-harm and the patient’s splitting behaviour. Tensions have arisen among staff; some team members consider the treatment purposeful, while others question the rationale for continued HIC admissions. A unified approach within the (treatment) team is lacking. The patient has previously been placed in seclusion, but this has proven counterproductive, making de-escalation of care nearly impossible. As a result, one-to-one supervision has been maintained for several weeks. Care providers report feeling at the end of their tether and express a deep sense of helplessness. They are reluctant to reduce the level of care due to the ongoing risk of self-harm and suicidality. The outpatient team has stated that they are unable to provide care in the home environment and do not feel able to take on the responsibility.

### 4.2. Focus Groups

In Phase 2, the two vignettes were discussed in the focus groups. Part A summarises participants’ reflections on each vignette, including perceived impact on the team, doubts and dilemmas surrounding admission, and proposed management strategies. Part B delineates two overarching themes that emerged from the focus groups: enhancing team communication and clinical decision making, and strengthening partnerships and shared responsibility. Throughout these reflections, problems at the level of the patient, the team, the ward and the wider system emerged as closely intertwined.

**Part A. Vignette-Specific Findings**
**Vignette 1**
**Impact On The Team**

Participants widely recognised Vignette 1, emphasising that the combination of ongoing threat, unpredictability, and lack of perspective placed the team under sustained pressure. Constant alertness undermined their sense of safety and well-being, compromised adherence to the HIC model, and led to increased use of coercive measures. Over time, this tension eroded empathy and objectivity, especially after serious incidents. Participants attributed their sense of powerlessness to multiple factors outlined in the vignette. When reflecting specifically on their own role, they mentioned limited expertise in addiction and intellectual disability (ID) care, as well as difficulties managing highly aggressive and unpredictable behaviour. Consequently, team morale and cohesion suffered, sometimes resulting in staff sick leave.

**Doubts and Dilemmas Surrounding Admission**

Participants were divided on whether Case 1 should be admitted to an HIC ward, raising several distinct concerns. First, many questioned whether HIC was the appropriate setting for patients with predominant antisocial behaviour and substance use, suggesting that addiction services, social work, disability, or forensic care would be more suitable. However, systemic barriers, such as cost-driven policies, insurer decisions, and legal procedures, often hindered effective collaboration and transfer, especially for patients in a ‘grey area’ without formal forensic status. Despite these doubts, participants felt pressured by society, chain partners, and their own management or psychiatrists to admit such patients:

Nurse: “It has to go wrong here before the patient ends up in the right circuit, at least forensic, and that comes at the expense of everyone. He doesn’t get the care he needs, and someone becomes a victim, or several people do.“

Psychiatrist: “It remains a fact that it is just a grey area between forensic care and mental health care. [...] I am always discussing with my colleagues from the forensic departments: ‘This is more for forensic,’ and they say, ‘But he does not have the title, so it is not forensic.’ [...] It would help if, across mental health care, we had more feeling for where that boundary actually lies. For this case, I would say forensic department first.“

Second, participants noted a lack of treatment perspective, which was closely linked to the absence of clear treatment goals and plans. Limited patient insight and motivation, combined with unclear objectives, often resulted in prolonged admissions, frustration among staff, and an increased risk of aggression and coercive measures. Third, significant issues with patient flow were reported. The lack of concrete treatment plans and goals complicated discharge planning and transition to other services. Additional barriers included unprepared discharges by network partners, reluctance from outpatient or forensic care providers to accept patients, a shortage of suitable placements, and long waiting lists. These factors collectively contributed to stagnation and inefficiency in the care process. Fourth, participants expressed frustration about a broader societal tendency to hold mental health services responsible for all disturbed and aggressive individuals. They felt that the police, by often ignoring or dismissing incidents on the HIC, reinforced this perception and contributed to the normalisation of aggression as part of psychiatric care:

Psychiatrist: “It sometimes feels as though, as a HIC, we are not truly part of society, as if we exist outside its boundaries.”

Nurse: “Yes, the police often say, ‘Well, that [aggression] is your job; you just have to deal with it.’ […] And I also notice that psychiatric behaviour is sometimes met with a tacit acceptance of criminal behaviour, as if being a psychiatric patient means you are permitted to do anything.”

**Proposed Approach: Adopting a Reward-Based Care System**

Despite these concerns, most participants were open to admitting patients as described in Vignette 1 if strict conditions were met. They advocated for a reward-based care system combined with room programmes to minimize triggers, emphasising structure, predictability, and positive reinforcement. As patients demonstrated appropriate behaviour, they would gradually earn more freedom. As one nurse explained:

Nurse: “The message is that we want to prevent things from going wrong, especially for their [the patients’] sake. Our aim is to help them [patients] experience success, rather than punishment or feelings of rejection and failure, as if he’s not good enough.”

Coordinating nurse: “Absolutely, that’s important for everyone. We all need positive reinforcement.”

This approach was seen as highly intensive, requiring strong team commitment, collaboration, specialised training, and facilities such as an available ICU and HSR, as well as the ability to quickly increase staffing levels. Participants also emphasised the need for respite options, such as a “carousel” system for temporarily exchanging patients between institutions. Team safety remained a central consideration throughout.

**Vignette 2**
**Impact On The Team**

Vignette 2 was also recognised as particularly challenging. Participants described a polarising dynamic within teams, with some staff favouring an autonomy-promoting approach, encouraging patients to take responsibility for their own care, while others preferred a more hands-on stance, taking over care and responsibilities out of a desire to help, or, in the case of clinicians, turning these cases into treatment projects, as illustrated by one nurse:

*“And I notice with clinicians that these cases become a sort of projects for them. Patients evoke this: ‘I cannot carry the responsibility, so you will.’ You feel some compassion, and clinicians say: ‘Fine, we will take over,’ while the whole team shouts: don’t do it, don’t do it.”*

This polarising split often led to conflict, inconsistency in treatment, and team exhaustion. Repeated suicide attempts in this patient group were described as traumatizing, further adding to the emotional burden. Although most participants experienced splitting as problematic, some saw them as an opportunity for open discussion and improvement.

**Doubts and Dilemmas Surrounding Admission**

Participants expressed fewer doubts about HIC admission in Vignette 2 but still raised three important concerns. First, there was a lack of consensus on addressing ‘acting out’ behaviours, such as self-harm and suicide attempts. Some saw these as evidence that admission was ineffective, advocating for discharge, while others supported a therapeutic approach focused on building trust and coping strategies. The peer worker observed that this internal division left patients feeling uncertain and insecure about their treatment, a situation attributed to limited staff expertise. Second, prolonged admissions were common, due to unclear treatment goals and insufficient follow-up services. Outpatient teams often refused to resume care, lacking capacity and expertise for high suicide risk. Inpatient teams also struggled to scale down care and move toward discharge:

*“We, the care providers, have created this situation: we cannot take her off one-on-one supervision now, because she will attempt to strangle herself. We have already found her unconscious several times, and she knows that, without supervision, she will succeed, and that is the only thing she wants at this moment. Although there are a few prospects, we have not been able to gain enough traction with her.”*

Nurse

Third, participants described how a lack of effective collaboration with network partners, particularly the police, complicated crisis planning and often resulted in repeated court-ordered admissions, situations they felt rarely benefited the patient and placed additional strain on the HIC.

**Proposed Approach: Implementing an Autonomy-promoting policy**

Despite the challenges, participants saw potential for HIC admission using an autonomy-promoting policy, involving short-term, voluntary time-out admissions focused on restoring patient control and avoiding seclusion or escalation. One-on-one supervision was discouraged, as it rarely led to progress. Accepting inherent risks and investing in the therapeutic relationship were considered essential:

*“We do not use camera surveillance, holding, or an HSR. There are situations where someone may attempt to strangle themselves twenty times a day, and you have to intervene each time. Still, you keep investing in the relationship and making contact: ‘We start again, we appeal to the healthy part of you that is still there, let us know what you need from us […].’ Then you have to walk away from the situation, as contradictory as it may feel from your nursing heart. But ultimately, if the whole team is committed to this approach, you can truly break the cycle and achieve significant success.”*

Nurse

The intensity of these admissions required regular team and peer coaching, clear treatment plans, and strong network support. During admission, no medication changes or extra care should be provided; these must be arranged externally.

**Part B. Overarching Findings Relevant to challenging Situations**
**Enhancing Team Communication and Clinical Decision-Making**

Participants noted that effective team involvement is often lacking in complex admissions. They emphasised the need for a safe, open culture, clear alignment, and timely decisions. Delays or unclear policies left staff feeling unsupported and unsure how to proceed. However, a positive shift toward more decisive action and appropriate risk-taking was noted, leading to quicker placement in suitable settings:

*“I’m very glad that cases like this [Vignette 2] no longer come to my ward, because we have very decisive psychiatrists. [...] They’re willing to take risks, put it in writing, and communicate it clearly. There are clear boundaries: a maximum of five days or straight to an open ward, crisis measures lifted immediately, and as much freedom as possible is restored and communicated. Family is involved in risk policy. Our psychiatrists have a clear stance, and it’s supported by management.”*

Nurse

Regular team meetings to discuss challenging situations, team training, and clear treatment and emergency plans were recommended to support decision-making. Conversely, teams should keep management informed to enable timely action, and psychiatrists should consult each other more often to ensure consistency and support.

**Strengthening Partnerships and Shared responsibility**

All participants agreed that complex cases require shared responsibility among network partners, including police, municipality, outpatient care, and family. Police and other partners often failed to take responsibility, instead directing cases to the HIC. Clear agreements on roles and practical arrangements, such as shared emergency plans, were seen as key to shifting accountability:

*“I think the key is to stick to what you’re good at. We’re good at psychiatry, so that’s what we do. The rest falls outside our responsibility and sometimes this leads to friction with the police or justice system. [...] It requires a lot of coordination and clarity: we’ve achieved our goal, so as far as we’re concerned, the admission is finished. We’re willing to think along with our network partners, but how are we going to do this together?”*

Nurse

Participants recommended developing a shared vision and clearer guidelines within psychiatry for ‘grey area’ cases, prioritizing staff safety. They valued effective communication with the public prosecutor and a “two-track policy” combining legal action with psychiatric care. The Safety House, a cross-sector regional network for complex cases, and Intensive Home Treatment (IHT) teams were seen as helpful for smoother admissions and discharges. Finally, joint training and stronger connections between sectors were advised to support good decision-making.

## 5. Discussion

**Figure 1:**
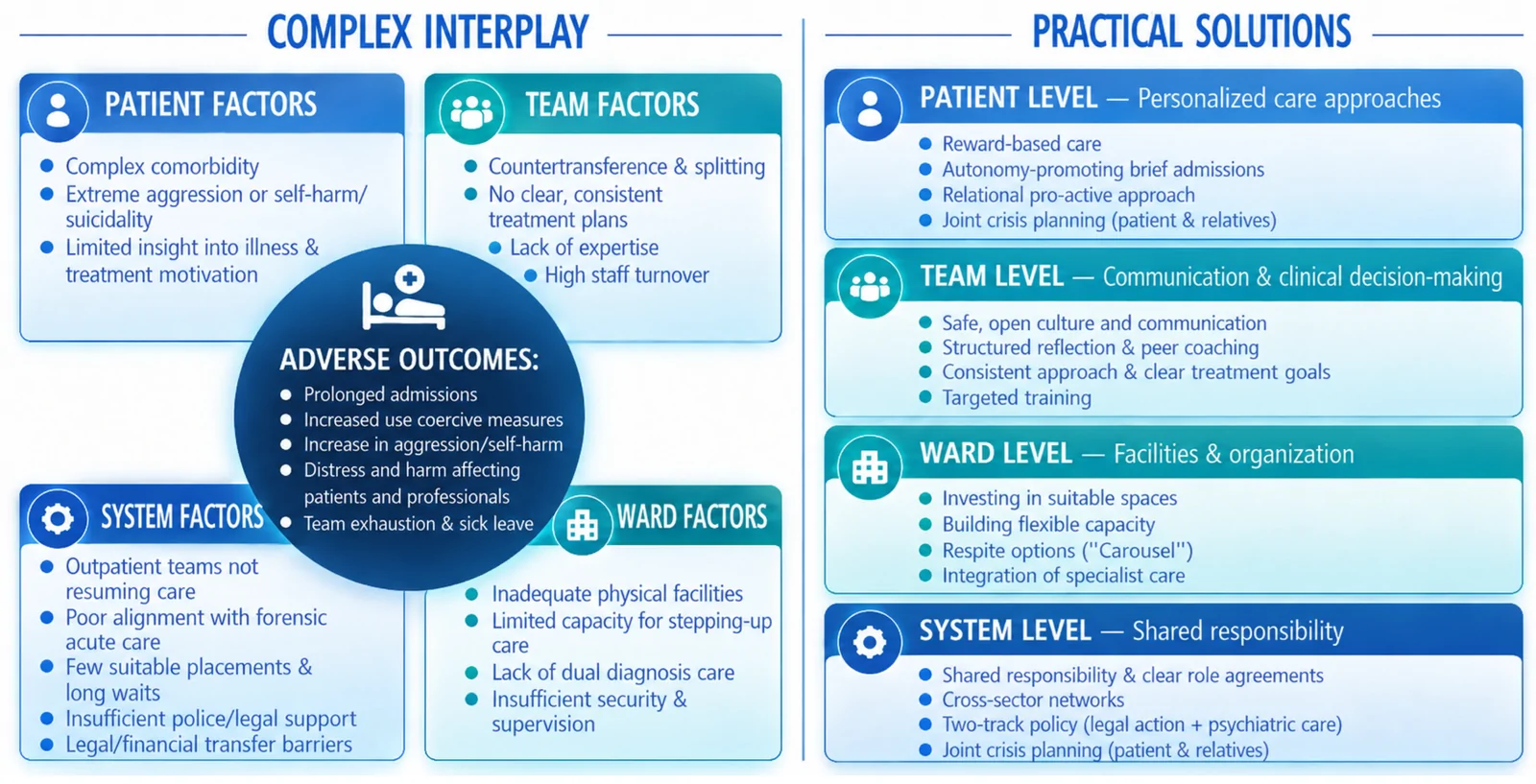
Complex interplay of patient, team, ward, and system factors leading to adverse outcomes in challenging situations on HIC wards, and the levels at which proposed solutions operate.

Through case analyses, vignettes, and focus groups, two common challenging situations were identified. Both were universally recognised by participants as difficult and accompanied by doubts and dilemmas. The patient, team, ward, and system factors involved largely correspond to those described in the literature [2,3,7], although ward and system factors have received less attention. Crucially, however, it was not these factors in isolation, but their complex interplay, that made care difficult and persistent. Earlier studies described these factors as separate contributors to difficult situations [2], as circular patient–professional interaction patterns maintaining refractory cases [3], or as an interplay of challenges experienced by nurses [7]. The present study shows how factors at all levels converge in specific challenging situations. It also reveals which established approaches teams themselves consider applicable. Figure 1 schematically depicts how the interplay between these various factors leads to adverse outcomes, and the solutions proposed at each level. In the following sections, this interplay and these solutions are further elaborated and discussed. First, the teams’ responses to the challenging situations are explored. Then, the proposed solutions are critically examined.

### 5.1. Dealing with Countertransference and Splitting

Our study confirmed that certain patient groups trigger strong emotional responses, such as anger, frustration, anxiety, and helplessness, within care teams, as previously described [14,19,20]. Persistent threat and unpredictability undermine core HIC principles like empathy and attentiveness, a finding supported by the literature [14,19]. This often manifests as countertransference, which refers to staff’s emotional reactions towards patients shaped by their own vulnerabilities [20]. Another manifestation was splitting, the formation of opposing groups with divergent views on policy and feelings toward the patient. According to Green [19], this dynamic is shaped both by patient behaviour and team characteristics and happens particularly in cases involving patients with personality disorders and suicidal behaviour. Both splitting and countertransference intensify tension and complicate collaboration, not only among health care professionals but also with patients, as confirmed by previous research [19,20]. These dynamics frequently provoke dismissive responses from care teams, such as neglect, or considering transfer or discharge, which can reinforce feelings of rejection in patients who are in urgent need of care [15,21]. Importantly, our findings, together with the existing literature [14,19], highlight that, despite their challenges, these dynamics also offer opportunities: timely recognition and open, blame-free discussion are essential for improving collaboration and fostering self-reflection within care teams.

### 5.2. Balancing Safety and Promoting Understanding Approaches

Although many empathic approaches were discussed in the focus groups, our study found that professionals frequently resorted to more controlling strategies, such as extended supervision, room restrictions, or the use of the HSR, when faced with escalating situations. While these interventions may appear logical in moments when staff feel unsafe or lose their sense of control [14], our findings indicate that they provided only short-lived relief and contributed to adverse outcomes, including escalation of aggression and self-harm, coercive measures, and prolonged admissions, consistent with reports of poorer outcomes and more frequent readmissions [22]. However, research consistently demonstrates that such challenging behaviour often reflects unmet needs, particularly when underlying emotional issues remain unaddressed by staff [4]. Humane care and ethical sensitivity are central to managing challenging behaviour; as such, approaches that focus on patients’ emotional and psychological needs tend to be more effective and provide more lasting benefits [2,14,15], even when such strategies may initially feel counterintuitive [3]. Rather than viewing (self-)aggressive behaviour as merely disruptive, it should be recognised as a signal of serious self-regulation difficulties, an area where nurses and patients can work on together to achieve meaningful change [21]. Building on Terkelsen and Larsen’s [14] recommendation to prioritise prevention, a view echoed in our own findings, clinical practice should focus on proactive strategies, such as reducing triggers, fostering empathy, engaging in patient-driven dialogue, and adopting a non-stereotyping approach. Furthermore, evidence shows that well-trained and supported staff, working within a positive and safe team culture, are more likely to adopt empathic and communicative approaches and less likely to resort to controlling or coercive measures [5,23]. To support staff competence in behaviour management throughout the care process, the comprehensive conceptual framework developed by Tölli et al. [4] may offer valuable guidance in identifying the necessary knowledge, skills, and safety aspects for effectively dealing with challenging situations. Investment in staff training, ongoing support, and the cultivation of a healthy team environment is therefore essential to promote safe, humane, and effective care.

### 5.3. Cross-Sector Collaboration and Mutual Learning

Consistent with earlier research [2,12,24], challenging situations on HIC wards require team-based approaches to support and educate professionals. Our findings add that teams themselves see a role for reward-based care in situations such as that described in Vignette 1. Participants considered this approach valuable because it offers structure and predictability, allows patients to gradually regain freedom as they demonstrate appropriate behaviour, and helps them experience success rather than punishment or rejection, while team safety remained a central consideration throughout. Reward-based care, also known as token economy, reinforces positive behaviour and is well established in ID care. This field is highly relevant to HIC: much of the research on challenging situations and restrictive interventions originates there, and patients with MID feature prominently in the challenging situations encountered on HIC wards. Reward-based care has proven effective in reducing aggression in various settings, including psychiatric hospitals [25,26]. However, our findings indicated that applying this approach in the HIC setting is highly demanding, as it requires strong team commitment, specialised training, appropriate physical facilities, and the ability to rapidly increase staffing levels; conditions that are not met on every ward. This is consistent with the literature, which similarly emphasises that implementing reward-based care requires specialist training and close collaboration with multidisciplinary teams and families [18,27].

While some participants maintained that HIC wards are unsuitable for this patient group and argued for transfer to forensic wards with higher security levels, it remains debatable whether such placements truly address the underlying challenges or merely shift them elsewhere. The findings of this study, together with the existing literature [15,28,29], suggest that many of these challenges can be addressed by enhancing meaningful engagement, addressing the root causes of disruptive behaviour, and implementing alternative strategies, such as those proposed here. Moreover, transferring patients to forensic care is a complex process, involving significant organisational and legal barriers, as confirmed in this study, and is not always justified or in the patient’s best interest [30]. At the same time, closer collaboration and shared learning with forensic services as well as with other sectors, such as ID care and addiction services, can help HIC teams access a wider range of expertise and practical approaches. In some cases, temporary transfer to trans-forensic or forensic units, like the Forensic High and Intensive Care (FHIC), may be appropriate [30,31]. However, further research is needed to better define the ‘grey area’ patient and to determine when such transfers are truly warranted. Ultimately, fostering a culture of mutual learning and partnership across disciplines is essential for delivering safer and more effective care for patients with complex needs.

### 5.4. Balancing Autonomy and Active Professional Support

Consistent with recent research and updated clinical guidelines [32,33], our findings underline the value of voluntary, brief, non-restrictive admissions with clear goals and joint crisis planning for individuals with personality disorders and suicidal behaviour. In Vignette 2, participants proposed such an autonomy-promoting policy, involving short-term, voluntary time-out admissions focused on restoring patient control and avoiding seclusion or escalation. What our study adds is that teams themselves consider these approaches applicable to the specific challenging situations encountered on HIC wards. Although brief admissions are valued for promoting autonomy and self-esteem, a review of the literature and our findings reveal that the specific content of care during these admissions remains largely undefined. In particular, there is little guidance on how to manage challenging behaviours, such as acting out, self-harm, or suicide attempts, during brief admissions. This lack of clarity creates a tension between two extremes, as seen in both our study and the literature: on one hand, an overly controlling approach persists, as nurses often find it difficult and stressful to relinquish control to patients at risk of self-harm or suicide [34]; on the other hand, a strong focus on autonomy can result in a passive or distant professional attitude, leaving patients feeling unsupported or even denied appropriate care due to stigma and unnuanced attitudes [21]. Prior research on patient-reported experiences shows that inpatient stays are frequently perceived as impersonal, repetitive and lacking clear direction, with repeated pharmacological interventions exacerbating feelings of hopelessness and dependence on the system [35]. Patients themselves value being listened to, having meaningful conversations, and experiencing a sense of safety and control [36], while professionals must also maintain clear boundaries to prevent excessive emotional involvement [37]. To improve outcomes and to prevent the risk of inaction, several studies suggest that brief admissions should include additional interventions, such as individualised programmes or structured activities [33]. In addition, embracing relational autonomy can help bridge the gap between overly controlling and overly distant approaches by encouraging professionals to actively engage with patients as vulnerable individuals, supporting their autonomy within a trusting relationship while still taking responsibility for their care needs [38]. For clinical practice, brief, voluntary, and non-restrictive admissions may therefore be considered for individuals with personality disorders and suicidal behaviour, supported by clear goals, shared crisis planning, and a relational approach that ensures the high quality specialist care expected in HIC settings. However, participants emphasised that implementing an autonomy-promoting policy in HIC settings is challenging, as it requires regular team and peer coaching, clear treatment plans, and strong network support, while division within the team and limited expertise can hinder a consistent approach. Future research should therefore define the core components of care during these admissions, develop strategies for managing challenging behaviours, and examine how relational autonomy and structured interventions can be effectively embedded in specialist services. At the same time, we would like to stress the importance of these approaches for daily practice, and we see a clear role for the nursing teams themselves in giving further shape to their implementation, drawing on the existing protocols and literature in which these methods are described.

### 5.5. Implications for Mental Health Nursing Practice

Challenging situations in HIC wards often involve patients with complex comorbidity, namely personality disorders and suicidal behaviour, or ID and aggression, combined with team, ward and system factors. As nurses provide continuous care and are most directly exposed to these situations, they are particularly affected by the emotional dynamics involved, such as countertransference and splitting. Timely recognition, open discussion, joint reflection, staff training, and nursing involvement in decision making are therefore essential. Moreover, the approaches proposed by participants, reward-based care and autonomy-promoting policies, are largely nursing interventions, requiring nursing expertise, consistency, and strong team commitment. Nursing teams thus have a clear role in giving these approaches further shape in daily practice, drawing on existing protocols and cross-sector collaboration. Given the exploratory nature of this study, these approaches should be regarded as promising directions rather than evidence-based interventions for HIC settings; their implementation depends on the conditions described above and should be accompanied by careful evaluation. Personalised, proactive, and empathic approaches, supported by clear protocols and cross-sector collaboration, can address patient needs, enhance staff safety, and reduce controlling responses. Participants in this study identified these approaches as key, drawing on their practical experience. Strengthened collaboration and further evaluation of recommended interventions are needed for sustainable acute psychiatric care.

### 5.6. Strengths and Limitations

It should first be noted that the vignettes used in this study are based on real-life cases from 20 different HIC wards, and we found a striking degree of similarity in the key features of difficult situations across these settings. As such, the vignettes reflect the most common and salient patterns of complexity encountered specifically within the HIC context. However, in other contexts or care settings, different types of difficult situations may be more prominent. For example, previous research [3] identified self-neglecting patients as a particularly challenging group, which may be more characteristic of ambulatory or community-based care. The vignettes in this study are therefore intended to facilitate discussion about complex cases in HIC, but do not represent an exhaustive typology of all possible challenges in acute psychiatric care. It would be valuable for future research to replicate this approach in other settings to explore whether similar or different patterns of complexity emerge. Beyond this, a key strength of this study is the in-depth exploration of case examples, which allowed for identification of specific situations which disrupt team dynamics and challenge the core principles of HIC. The involvement of many institutions in case retrieval and focus groups enabled valuable knowledge sharing and cross-institutional learning. Teams contributed unique insights based on their practices, policies, and collaboration with external partners, reflecting diverse organisational contexts. This variety strengthened our findings and increased their relevance and applicability to national trends.

Nevertheless, some limitations should be acknowledged. A limitation related to the case material is that some of the cases submitted lacked detail, which may have limited the depth of analysis. In retrospect, providing more specific instructions for the case inquiry might have improved the consistency and richness of the submitted cases. Moreover, the wards themselves selected which cases to submit, which may have introduced selection bias: the two identified types represent the most common patterns within this sample rather than a definitive typology, and other challenging situations may be underrepresented. By design, the vignettes describe situations in which care was particularly challenging; participants proposed and reflected on solutions, but these were not evaluated in successful cases. A valuable complement could be to include successful cases in future research, examining whether these approaches actually work and which factors contribute to their success. With regard to the focus groups, because HICs were allowed to select their own representatives, not all professional roles present on HIC wards were equally represented, and some disciplines were more prominent than others. However, this distribution also reflects the actual composition and dynamics of HIC teams in practice. Notably, we did not observe clear differences in perspectives or opinions between the various professional roles, for example, nurses and psychiatrists, represented. Patients, family members, as well as network partners were not included in this study, which means that the situations and proposed approaches are described exclusively from the perspective of HIC professionals. Although their perspectives would have been valuable, this was not the focus of the current study; future research should involve these groups for a more comprehensive understanding. While the focus groups did not allow for in-depth exploration of all concepts, especially systemic issues, they did reveal what makes these situations specifically challenging in the HIC context, as well as concrete approaches and conditions for managing them.

## 6. Conclusions

This study systematically identified and explored the most challenging situations faced by inpatient care teams at HIC wards. Two recurring types of challenging situations emerged, each marked by complex interactions between patient, team, ward and system factors and both recognised as highly demanding by professionals. These cases often evoke strong emotional responses and can lead to counterproductive team dynamics such as splitting and countertransference. Recognising and understanding the specific combinations of patient and contextual factors in challenging situations is the first essential step to subsequently anticipate, identify, and respond to these complex situations. Our findings highlight that dismissive or controlling responses, while understandable, are rarely effective and may reinforce negative outcomes. Instead, individualised, proactive, and empathic approaches, supported by clear protocols and ongoing team reflection, are essential for safe and effective care. The study also underscores the importance of cross-sector collaboration and mutual learning, particularly in managing aggressive behaviour and complex admissions. Brief admissions, while promising for promoting autonomy, require clearer guidance and additional interventions to address challenging behaviours and avoid extremes of control or neglect. Investing in staff training, structured support, and interprofessional collaboration is crucial. Future research should further define criteria for complex admissions, evaluate the effectiveness of specific interventions, and include patient and network partner perspectives to ensure comprehensive, sustainable solutions for acute psychiatric care.

## Figures and Tables

**Table 1:** Question framework for online case collection.

**Questions**
Case 1 Could you describe a case involving a patient where a challenging situation arose prior to admission, in which it was stated: “We cannot admit this patient to the HIC *”? Please describe the situation (without names, date of birth, or institution name): - What were relevant patient-related factors, and why were they relevant? - Which factors played a role at the team or institutional level, and why? - Where did you perceive the main difficulties, and why? - What do you think would have been best for the patient in question, and what would you have needed to achieve that? Please explain why. Case 2 Could you describe a case involving a patient where a challenging situation arose during admission, in which it was stated: “We cannot keep this patient at the HIC *”? Please describe the situation (without names, date of birth, or institution name): - What were relevant patient-related factors, and why were they relevant? - Which factors played a role at the team or institutional level, and why? - Where did you perceive the main difficulties, and why? - What do you think would have been best for the patient in question, and what would you have needed to achieve that? Please explain why.

* HIC: High and Intensive Care.

**Table 2:** Topic list for the focus groups.

**Questions and Prompts**
- Is this case familiar to you? - What stands out to you? - Is there anything missing? - What makes this case particularly challenging for you? - Why is this situation especially challenging for an HIC * ward? - What are the pain points? - Why is it not working in this situation? - Is it a matter of lacking resources at this moment? - Is this patient fundamentally unsuitable for admission to the ward? - Is the HIC * intended to accommodate patients like this? - Where do you draw the line, what makes one case more intense than another? - How do you generally deal with situations like this, and what has worked well? - What are you currently doing to manage this type of situation? - What would be the best way to handle this, and what do you need to do so? - What does this mean for your ward, and for HIC * care more broadly? - What is the opinion of the coordinating clinician? Policy may be strongly influenced by this. - Do you notice differences in how this is experienced across professional roles? - Whose responsibility is this? - Where should these patients go instead? - Is this about a difficult patient or a challenging situation? - *(For Vignette 2)* Is personality disorder a contraindication for acute inpatient admission longer than one day?

* HIC: High and Intensive Care.

## Data Availability

The data that support the findings of this study are available from the participating mental health institutions. Restrictions apply to the availability of these data, which were used under licence for this study. Data are available from the authors with the permission of the participating mental health institutions.

## Supplementary Materials

The following supporting information can be downloaded at: https://www.mdpi.com/article/10.3390/nursrep16100356/s1, Supplementary Table S1: Case collection organised by case number, gender, age, clinical background, patient-related factors, team-related factors and ward-/system-related factors; grouped in two vignettes. https://www.mdpi.com/article/doi/s1, Supplementary Material S1: Completed COREQ (Consolidated Criteria for Reporting Qualitative Research) Checklist. Reference [39] is cited in the Supplementary Materials.

## Public Involvement Statement

In accordance with the GRIPP2 guidelines, we report that patients or members of the general public were not involved in the design, conduct, or dissemination of this study. Consultation and input were strictly limited to the professional healthcare staff working within the High and Intensive Care (HIC) units. The HIC healthcare staff acted solely as study participants rather than active research partners.

## Guidelines and Standards Statement

This study was conducted and reported in line with the COREQ (Consolidated Criteria for Reporting Qualitative Research) guidelines and a completed COREQ checklist has been added as Supplementary Material (S1).

## Abbreviations

The following abbreviations are used in this manuscript:

HIC	High and Intensive Care
PTSD	Post-Traumatic Stress Disorder
FACT	Flexible Assertive Community Treatment
IHT	Intensive Home Treatment
HC	High Care
IC	Intensive Care
ICU	Intensive Care Unit
HSR	High Security Room
MID	Mild Intellectual Disability
nWMO	niet onder de Wet Medisch-wetenschappelijk Onderzoek (not under the Dutch Medical Research Involving Human Subjects Act)
