# Supplementary material for: Challenging Situations in High and Intensive Psychiatric Care: Professional Perspectives and Key Characteristics
Source: Nurs Rep. 2026 Oct 9;16(10):356. doi: 10.3390/nursrep16100356 (PMC13708545; doi:10.3390/nursrep16100356)
Supplement: Supplementary file 1 [file nursrep-16-00356-s001.zip › Supplementary Table S1.pdf]

Supplementary material

Table S1. Case collection organised by case number, gender, age, clinical background, patient-related factors, team-related factors and ward-/system-related factors; grouped in two vignettes.

Case number	Gender	Age range	Clinical Background/ Characteristics	Patient-related factors	Team-related factors	Ward-/system-related factors
			Vignette 1			
1A	M	35-40	Psychotic vulnerability related to chronic polysubstance use; antisocial personality traits; long history in SUD care; no stable housing.	Recurrent life-threatening intoxication and substance-induced destabilisation; boundary-violating, group-disrupting behaviour; limited recovery perspective.	Strong countertransference due to repeated failures; disagreement about treatment options; fear and frustration around risk.	Step-down from high-security to HIC too abrupt; ward not equipped to combine high security with intensive SUD care.
1B	F	40-45	Schizophrenia; MID; long forensic psychiatric history; frequent severe incidents.	High aggression risk related to psychotic symptoms and ID; limited understanding of ward structure; absence of social network.	High anxiety about safety; limited collaboration with external partners; doubts about usefulness of brief crisis admission.	HIC setting lacks adapted structure and security for acute psychosis in a person with ID and forensic history.
2A	M	40-45	Psychotic disorder; SUD; history of attempt to strangle staff; frequent offending behaviour.	Persistent psychotic symptoms with marked aggression; limited insight and impulse control.	Staff fear due to prior serious assaults; limited support from forensic system; moral distress about being asked to take "too dangerous" patient.	No 24/7 High Security beds; HIC infrastructure and staffing insufficient for this violence risk.
3A	F	-	Trauma-related disorder; psychotic vulnerability; severe SUD;	Recurrent substance-related manic-psychotic symptoms; care avoidance after	Countertransference from many short admissions; sense of futility around SUD; new staff feel unsafe	HIC can stabilise acute episodes but lacks dual-diagnosis crisis and SUD treatment capacity;

Case number	Gender	Age range	Clinical Background/ Characteristics	Patient-related factors	Team-related factors	Ward-/system-related factors
			unstable housing and support system.	discharge; repeated re-engagement with high-risk environments.	due to sexual disinhibition/aggression.	structural gaps in SUD services.
3B	M	-	Unspecified psychotic disorder with manic symptoms; cluster B personality traits; substance use (cocaine); language/cultural barriers.	Impulsive aggression and sexually disinhibited behaviour; limited history and communication complicate assessment; fluctuating illness insight.	Limited forensic and personality-disorder expertise; splitting; high insecurity and fear.	HSR/HIC environment insufficiently secure for prolonged complex high-risk care; weak police collaboration; no straightforward forensic pathway.
4A	F	35-40	BSD with psychotic traits; antisocial personality disorder; early attachment trauma; extensive forensic history.	Severe emotion-regulation problems with aggression and stalking; chronic group unsuitability; strong autonomy needs.	Countertransference due to long familiarity; staff included in delusional system; difficulty offering equal opportunities compared to other patients.	Open HIC ward offers too little containment; need for smaller, more structured unit with clearer boundaries and security.
4B	M	30-35	Psychotic symptoms with persecutory delusions; polysubstance use; recent period of incarceration; MID; extensive psychiatric and forensic history.	Bizarre and changing psychotic symptoms after unclear substance use; over-asking due to ID; limited illness insight.	Incomplete information as guest placement; multiple duty doctors, no unified policy; uncertainty about intoxication vs withdrawal; limited knowledge of GHB withdrawal.	HIC lacked routine somatic/toxicology screening; somatic intensive care ultimately required but initially overlooked.
5B	M	25-30	Disruptive/impulse-control disorder; SUD	Limited social network; institutionalisatio	HIC must hold patient without clear goal; risk of frustration and	Supported living withdraws without transparency; HIC is

Case number	Gender	Age range	Clinical Background/ Characteristics	Patient-related factors	Team-related factors	Ward-/system-related factors
			(cannabis); history of severe aggression during past admissions; long hospitalisation; clozapine treatment.	n; housing and financial problems; dependency on one supported-living provider.	possible aggression due to lack of perspective.	used as long-stay placement without treatment target; no binding agreements with downstream services; few alternative housing options.
6A	M	-	Schizoaffective disorder (bipolar type); ID; impuls-control difficulties; cannabis use; post-anoxic brain injury; frequent severe aggression and sexual boundary violations.	Limited response to medication; high impulsivity and aggression; primary need for structure and shelter rather than brief crisis treatment.	HIC staff feel under-skilled in ID crisis care; strong emotional burden and ethical concerns; need for shared plan.	Transmural team overburdened; HIC is designed for short psychiatric crises, not long-term high-risk containment; requires closed-room programme and high staffing beyond standard capacity.
7A	M	-	Schizophrenia with persistent psychotic symptoms; ASD; SUD; antisocial personality traits; history of multiple severe assaults.	Previous serious violence towards staff and others for which police reports were filed, but without judicial follow-up.; limited illness insight; difficulties accepting limits.	Staff feel unsafe and insufficiently supported by police/justice.	Limited amount of ICUs; insufficient security level for this patient category; lack of accessible forensic psychiatry beds.
7B	-	-	Severe behavioural disorder characterised by throwing objects and hitting staff; high care dependency; limited treatment	Persistent behavioural dysregulation; inability to care for self; minimal engagement in change-oriented interventions.	Team trained for brief crisis work feels overwhelmed by long-term high-intensity care; frustration and moral distress without improvement.	HIC is not a long-term high-dependency unit; infrastructure and staffing ratios do not support ongoing intensive care needs.

Case number	Gender	Age range	Clinical Background/ Characteristics	Patient-related factors	Team-related factors	Ward-/system-related factors
			response.			
8A	M	-	History of BSD diagnosis, later revised to antisocial personality disorder; recurrent aggressive, intimidating and manipulating behaviour.	Admission because of offending behaviour with minimal psychiatric findings; previous severe aggression incidents at ward.	Ward fears loss of control; anxiety about conflation of offending; lack of psychiatric presentation; poor pre-admission consultation with crisis team.	HIC used as substitute for justice system; no clear forensic pathway; ward's treatment mandate misaligned with primary risk (offending behaviour).
9A	M	-	Personality dysfunction with narcissistic personality traits; psychotic vulnerability; history of severe violence (e.g. neck holds on staff); physically strong.	Limited internal inhibition; poor tolerance of rules and hierarchy; high risk of targeted aggression; minimal insight into impact on others.	Persistent fear and trauma among staff; reluctance to confront or set limits; sense that forensic framework is required but unavailable.	HIC lacks graded security and specialised aggression-management infrastructure; standard ward cannot safely contain repeated severe violence.
10B	M	20-25	Psychotic disorder with manic symptoms; SUD (cannabis and nitrous oxide); no prior ambulant framework; complex family dynamics.	Severe substance-related decompensations; craving and limited motivation to stop use; repeated relapse; influenced strongly by family; boredom and frustration in HSR.	High staff strain from long 1-on-1/4-on-1; fear of renewed aggression.	Disrupted treatment alliance with patient and family; family undermines medication and boundaries; HIC lacks higher-security infrastructure; few meaningful activities possible in seclusion; limited rapid access to higher-security beds.
11B	M	-	Fetal alcohol spectrum disorder; BSD; antisocial personality traits;	Normalised extreme verbal aggression; recurrent boundary-	Inconsistent boundary-setting; some staff fearful, others minimising; uncertainty about how	Ward lacks clear behavioural protocols; inconsistent consequences

Case number	Gender	Age range	Clinical Background/ Characteristics	Patient-related factors	Team-related factors	Ward-/system-related factors
			psychotic symptoms; cannabis use; long history in foster care.	violating behaviour; ongoing substance use; limited recognition of impact on others.	to distinguish illness from maladaptive coping; difficulty forming unified behavioural plan.	undermine safety; HIC milieu becomes chronically hostile and destabilising.
12A	M	-	Personality dysfunction with narcissistic traits and psychotic symptoms; history of severe assaults (e.g. neck hold on staff); refusal of medication.	Strong need for control; poor tolerance for rules; limited illness insight; high risk of severe targeted aggression when frustrated.	Team has significant fear and distrust; reluctance to provide another chance; difficulty implementing standard HIC processes under high safety concerns.	HIC has only seclusion as escalation tool; lacks intermediate security and dedicated security staff; ward cannot safely manage repeated high-level aggression.
12B	F	-	Personality disorder; SUD; recurrent brief substance-induced psychoses; expelled from shelters/night hostels; no stable housing.	Patterns of boundary-testing interactions with staff; episodes of escalatory behaviour linked to re-admission; limited engagement with trauma or SUD treatment; low readiness for change.	Team experiences splitting, resistance and high frustration; difficulty engaging in constructive dialogue.	HIC repeatedly used as default shelter; admission reinforces boundary-testing behaviour; ward not designed for chronic behavioural admissions without acute psychiatric indication.
13A	F	-	Intellectual functioning in the borderline range; prior neuropsychological assessment (no longer current); severe attachment difficulties;	No psychotic or manic episode at forefront; impulse outbursts with verbal and physical aggression; self-harming behaviour.	Team expertise focused on psychosis and mood episodes, not MID/attachment-based problems; difficulty formulating adequate interventions; growing sense of “holding without progress”.	HIC configured for acute psychosis/mania, not for LVB-related and attachment-based behavioural problems; lack of access to appropriate MID facilities; prolonged

Case number	Gender	Age range	Clinical Background/ Characteristics	Patient-related factors	Team-related factors	Ward-/system-related factors
			impulse-control problems; self-harm.			stay without perspective burdens ward and patient.
13B	M	50-55	Psychotic decompensation likely related to substance use; no illness insight; normal to muscular build; extensive offending record for assaults; reported severe aggression towards police.	Threatening behaviour and high aggression risk; limited insight; psychotic and substance-related instability; complex risk profile between general and forensic psychiatry.	Ward receives transfer due to threats on first unit; staff feel unsafe after taser use on ward and repeated personal threats; psychiatrist avoids direct contact; staff unable to work due to fear.	HIC has security level 1 and limited capacity for very high-risk aggression; collaboration with forensic services slow and difficult; grey zone between forensic and regular crisis care, delaying placement on a more suitable, higher-security ward.
14A	F	20-25	Substance-induced psychotic disorder (flakka/ketamine); severe SUD; multiple short admissions and acute assessments; current long admission under IT; uninsured and without stable housing.	Repeated escalating agressive behaviour, with limited illness insight.	Staff resistance due to repeated aggression; sense of futility; limited capacity to address social determinants; difficulty coordinating psychiatric and SUD perspectives.	HIC focused on acute psychosis, not prolonged SUD treatment; no dual-diagnosis crisis unit; homelessness and lack of insurance drive repeated crises beyond ward's remit.
14B	M	30-35	Schizophrenia with persistent psychotic symptoms; polymaterial use disorder; long history across many wards and SUD services; 1.5 years on HIC.	Chronic psychosis and ongoing substance use; institutionalisation; limited engagement with care; repeated rejection by other services fuels hopelessness.	Team feels stuck providing custodial care without clear goal; demoralisation and lowered expectations; frustration at being "last resort".	HIC used as long-stay placement; ward not designed for this; absence of supported housing options allowing controlled use limits discharge pathways.

Case number	Gender	Age range	Clinical Background/ Characteristics	Patient-related factors	Team-related factors	Ward-/system-related factors
15A	F	-	BSD with psychotic traits; pregnancy (39+ weeks).	Manic-psychotic symptoms intensify due to delivery-related stress; inability to use antipsychotics; escalating verbal and physical aggression; high anxiety.	Staff experience strong emotional impact of secluding a highly pregnant woman; fear of chaotic delivery scenario; many external professionals but no clear decision-maker.	HIC lacks integrated perinatal psychiatric capacity; poor collaboration with obstetric services; ward environment not suited to combined obstetric–psychiatric high-risk care. Partner admitted elsewhere; planned delivery in home region cancelled.
16A	M	-	Chronic psychotic disorder; refuses medication; community disturbance through paranoid calls and knife collection; severe self-neglect and weight loss.	Persistent psychotic symptoms with significant risk; strong resistance to care; limited insight; poor self-care.	Staff feel unable to safely admit without HSR; insufficient experienced staff for high-risk coercive care; frustration that structural reforms limit secure options.	Reduced HSR capacity and increased acuity leave HIC without adequate secure infrastructure; ward cannot safely manage expected violence without additional resources.
17A	M	-	ID; psychotic vulnerability with catatonia when off medication; prior multiple severe aggression incidents; prior forensic care.	Severe aggression when approached by staff; accepts police dominance rather than healthcare staff; limited understanding and cooperation; high risk during restraint; difficulty maintaining medication adherence.	Team fear due to prior descriptions and strength; disagreement about feasibility of contact; distress over inability to provide basic care safely.	HSR lacks design for safe care (e.g. food hatch); corridors/doors unsuitable for multi-person interventions; ward physically unsuited to needs; no dedicated safety team available.
17B	M	-	ASD; long stay on another ward	Extreme dependence on	Many staff refuse to enter due to repeated	HIC and ICU physical/organisation

Case number	Gender	Age range	Clinical Background/ Characteristics	Patient-related factors	Team-related factors	Ward-/system-related factors
			with rigid daily routines; admitted to HIC for time-out.	strict structure; unknown HIC environment and staff; high anxiety and aggression when routine disrupted; limited adaptation capacity; expresses wish to resume familiar programme.	assaults; team shrinks; disagreements between wards and disciplines about policy and timing of return.	structure cannot reproduce his programme; delayed return to home ward prolongs inappropriate placement and risk.
18A	M	25-30	substance-induced psychotic disorder; SUD (cannabis and 3MMC); ID; history of excited delirium with severe aggression requiring police and ICU.	Poor understanding of vulnerability; severe SUD with minimal motivation for abstinence; recurrent excited delirium with high somatic risk.	Staff recognise SUD as primary but have limited tools; fear of repeated severe assaults; difficulty coordinating with SUD and justice systems.	Regular HIC wards lack infrastructure for triple diagnosis (ID, severe SUD, psychosis) and excited delirium; absence of on-site ICU and forensic-SUD capacity.
18B	M	30-35	Schizophrenia; suspected ASD; admitted under IT after serious property destruction; alternating cooperative and aggressive behaviour.	Psychotic symptoms with unpredictable aggression; agitation about IT; limited insight into illness and triggers; sensitivity to over-stimulation.	Team feels unsafe; lacks de-escalation tools beyond basic measures; holiday staffing reduces continuity; frustration over limited judicial follow-up.	HIC is noisy and overstimulating; no higher-security, lower-stimulus alternative; ward infrastructure not designed for complex, unpredictable aggression.
20B	-	-	Manic episode with psychotic traits; transfer from another region; voluntary admission, later IT decision.	Worsening disinhibited psychiatric presentation; no illness insight; group-disrupting behaviour on ICU; active resistance	Staff initially underestimate risk; hesitancy to intervene; stressful, traumatic experience when serious assault occurs.	IT decision-making took too long, allowing resistance to build in patient and finally escalation.

Case number	Gender	Age range	Clinical Background/ Characteristics	Patient-related factors	Team-related factors	Ward-/system-related factors
				to medication; no collaboration; high threat of aggression.		
Vignette 2						
2B	F	25-30	DID; trauma-related disorder; personality dysfunction with borderline traits; chronic suicidality.	Suicidality and regressiveness increase during admission; refusal of guideline-based ambulant treatment; strong dependency on intensive care.	Tension between ambulant team and other referrers; feelings of causing harm by admitting; pressure from family/GP for inpatient DID treatment.	HIC repeatedly used for suicidality in personality pathology despite evidence of harm; limited specialized ambulant personality-disorder network.
5A	M	40-45	Schizoaffective disorder; PTSD; longstanding mood and behavioural problems; repeated SAs; complex medication history.	Need for psychotherapy, clarity and trust, but mainly receives medication and containment; discouragement after repeated rejection by therapy units.	Disagreement between psychiatrist (psychosis focus) and nursing team (personality focus); nursing observations disqualified; tension and disempowerment.	Seclusion and ward structure used cyclically without clear therapeutic exit; HIC becomes custodial rather than recovery focused.
6B	Non Binary	20-25	PTSD; functional neurological symptoms; dissociative disorder; past BPD diagnosis; early-life trauma; repeated crisis admissions for suicidality and self-harm.	Chronic suicidality; dissociative episodes worsen with trauma treatment; worsening suicidal ideation during inpatient admission.	Countertransference and division over usefulness of admission; heavy use of IT and 1-on-1; staff anxiety about reducing support; sense of ineffectiveness.	HIC model (high support, low autonomy, acute focus) reinforces regression; limited organisational flexibility for autonomy-enhancing programme; absence of a clinically appropriate alternative care setting.
8B	F	-	Personality disorder with	Extreme self-harm and suicidal	Limited personality-disorder expertise;	Region lacks specialised long-term

Case number	Gender	Age range	Clinical Background/ Characteristics	Patient-related factors	Team-related factors	Ward-/system-related factors
			dependent traits and severe emotion-regulation difficulties; trauma-related problems; chronic suicidality; recent bereavement and involuntary child placement.	behaviour; low impulse control; destabilisation by child protection issues; high dependency on caregivers.	strong countertransference and feelings of helplessness; difficulty maintaining consistent policy; staff traumatised by repeated severe self-harm.	personality/trauma unit; HIC must provide intensive risk-focused care beyond its intended scope.
9B	F	-	Personality disorder; chronic suicidal thoughts; recurrent severe self-harm; prior frequent seclusion in other facilities.	Entrenched self-harm pattern reinforced by hospital admission; difficulty adhering to safety agreements; resistance to addressing underlying personality and social issues.	High emotional and practical burden; staff splitting and conflict; feelings of manipulation and exhaustion; delayed access to full history.	HIC functions as revolving door; environment inadvertently reinforces behaviour; lack of supported housing options.
10A	F	20-25	Complex PTSD; BPD; possible autism spectrum traits; history of eating disorder; extreme chronic suicidality; many IT admissions.	Habitual self-harm and suicidality; strong fear of abandonment; limited support network; diagnostic ambiguity; desire for trauma treatment within HIC setting.	Extensive use of IT without benefit; burnout and sickness among staff; differing diagnostic views; external pressure from authorities to admit.	HIC environment increases regression and self-harm; danger inside judged higher than outside; repeated non-beneficial admissions destabilise ward milieu.
11A	F	-	Personality disorder; high suicidality; HIC admissions with	High suicidal risk combined with non-disclosure of plans; strong	Nurses carry heavy emotional burden and fear of lethal SAs; intense sense of	HIC admission does not reduce risk and may increase it; ward structure assumes

Case number	Gender	Age range	Clinical Background/ Characteristics	Patient-related factors	Team-related factors	Ward-/system-related factors
			splitting and covert SAs using toxic beans.	splitting towards staff and between services; limited capacity for collaborative safety planning.	responsibility; difficulty trusting patient's communication.	collaborative crisis work which is absent here; lack of intensive community care alternatives.
15B	F	15-20	BPD; depressive disorder; eating disorder; PTSD; chronic and acute suicidality; recent EMDR; transition from youth to adult care.	Recurrent severe SAs increasing during admission; reduced autonomy; limited trust in treatment.	High-risk policy creates strong organisational risk; staff exhausted and traumatised; some sick-leave; team increasingly sceptical about benefit of admission.	HIC cannot reduce risk below community levels; admission appears to worsen clinical picture; long therapy waiting lists delay appropriate care; crisis outside region difficult to organise; complex family dynamics.
16B	F	-	ASD; borderline personality traits; eating disorder; PTSD; antisocial personality traits; chronic suicidality and self-harm; long multi-institution history.	Frequent severe crises and police involvement; limited response to prior treatments; strong dependency; repeated offending behaviour.	HIC must take full control (zero tolerance) contrary to recovery ethos; many rules produce staff anxiety; lack of trust due to past harm; high workload from 2-on-1 care.	HIC de facto becomes high-security, high-control ward for one patient; strain on capacity and ethos; external systems rely heavily on HIC as main solution. High burden on services and society.
19A	-	15-20	Personality development disturbance; multiple severe SAs; automutilation; psychogenic non-epileptic attacks; transferred from youth to adult MHS.	Chronic and acute suicidality with creative methods; aggression when SAs are interrupted; difficulty keeping agreements; complex multi-problem profile.	No shared organisational vision for such multi-problem high-risk patients; differing clinician views; staff feel abandoned and unsure; repeated critical incidents erode morale.	HIC short, medication-focused model inadequate; ward not structured for sustained high-risk relational work; no specialised multi-problem unit available.
19B	F	20-	Personality	Persistent severe	Numerous incidents	HIC offers brief

Case number	Gender	Age range	Clinical Background/ Characteristics	Patient-related factors	Team-related factors	Ward-/system-related factors
		25	disorder with borderline and avoidant traits; PTSD; recurrent depressive episodes; social anxiety; ADD; multiple severe SAs with resuscitations; suspected ABI.	suicidality and self-harm; mixed cognitive and behavioural difficulties; aggressive behaviour towards staff.	and injuries strain team; rotation of physicians/psychiatrists produces differing policies; staff feel overwhelmed and unsupported; limited capacity for collaborative safety plans.	containment and medication but lacks integrated psychotherapy; ward processes cannot sustain coherent long-term strategy; repeated admissions destabilise milieu.
20A	-	-	PTSD attachment difficulties; later ASD diagnosis; destructively suicidal behaviour.	Active suicidal behaviour; inability to make and keep safety agreements; limited engagement in changing current state; treatment of underlying disorders blocked by acute risk.	Ambulatory teams pass crisis to HIC; HIC experiences admission as non-helpful or harmful but is legally obliged to IT; frustration and moral conflict.	Ward dynamics adversely affected by repeated non-beneficial admissions; lack of shared cross-system crisis protocol; ambulant teams stuck; divergent in-hours vs out-of-hours responses.

Abbreviations:
ABI = Acquired Brain Injury
ADD = Attention Deficit Disorder
ASD = Autism Spectrum Disorder
BPD = Borderline Personality Disorder
BSD = Bipolar-Spectrum Disorder
DID = Dissociative Identity Disorder
GHB = Gamma-Hydroxybutyrate
GP = General Practitioner
HIC = High and Intensive Care
HSR = High Security Room
ICU = Intensive Care Unit
ID = Intellectual Disability
IT = Involuntary Treatment
MHS = Mental Health Services
MID = Mild Intellectual Disorder
PTSD = Post-Traumatic Stress Disorder
SA = Suicide Attempt
SUD = Substance Use Disorder
